# Mandibular Molar Distalization Using Interradicular Microimplants and Direct Anchorage: A Case Report

**DOI:** 10.7759/cureus.105909

**Published:** 2026-03-26

**Authors:** Fidele Nabbout, Zouhair Skaf, Ursula Abou Kheir, Aline Rabahi Nabbout

**Affiliations:** 1 Orthodontics, Faculty of Dental Medicine, Lebanese University, Beirut, LBN; 2 Pediatric Dentistry, Private Practice, Beirut, LBN

**Keywords:** class iii, direct anchorage, mechanics, microimplant, molar distalization

## Abstract

Management of skeletal Class III malocclusion using traditional orthodontic mechanics can be challenging because of limited anchorage control and undesirable reciprocal effects. However, the introduction of microimplants allowed a better control of tooth movement, reducing the need for surgical intervention in selected cases.

This case report illustrates the effectiveness of direct skeletal anchorage using interradicular microimplants for mandibular molar distalization in the treatment of a skeletal Class III malocclusion.

A female patient aged 19 years showing a mandibular prognathism and a normodivergent growth pattern underwent mandibular dentition distalization using a direct anchorage protocol supported by interradicular microimplants.

A distalization of the mandibular dentition and a controlled vertical dimension were achieved. The mandibular plane rotated in a counterclockwise pattern, allowing the closure of the anterior bite while maintaining proper inclination of the lower incisors and stable alveolar bone support.

Direct anchorage using interradicular microimplants represents an effective non-surgical strategy for camouflage treatment in selected skeletal Class III cases.

## Introduction

Class III occlusal relationship remains a complex condition encountered in orthodontics. Successful management requires a thorough assessment of skeletal relationships, dentoalveolar compensations, facial esthetics, and anatomical limitations such as alveolar bone thickness and periodontal characteristics [1].

Conventional orthodontic approaches for Class III correction frequently rely on extractions or intermaxillary mechanics. However, these techniques often lead to undesirable side effects, including anchorage loss, compromised vertical control, and dependence on patient cooperation [2].

The development of temporary anchorage devices (TADs) has considerably expanded treatment possibilities. By providing skeletal anchorage independent of the dentition, TADs enable more predictable tooth movement and improved biomechanical control. Consequently, orthodontic camouflage can be considered in selected mild-to-moderate Class III cases, particularly when patients decline orthognathic surgery [3].

Various protocols have been proposed for achieving mandibular molar distalization using skeletal anchorage. Microimplants may be inserted at different anatomical sites depending on bone availability and treatment mechanics. Among these locations, the region between the roots of the second premolar and the roots of the first molar is frequently selected due to favorable bone density, accessibility, and relatively low surgical invasiveness [4-6].

Despite the growing interest in this approach, limited clinical evidence exists regarding the outcomes of direct anchorage mandibular distalization using interradicular microimplants. Therefore, the objective of this case report is to evaluate the clinical effectiveness of this technique with particular attention to treatment efficiency, biomechanical control, anchorage stability, and skeletal adaptations.

## Case presentation

Diagnostic evaluation and etiology

**Figure 1:**
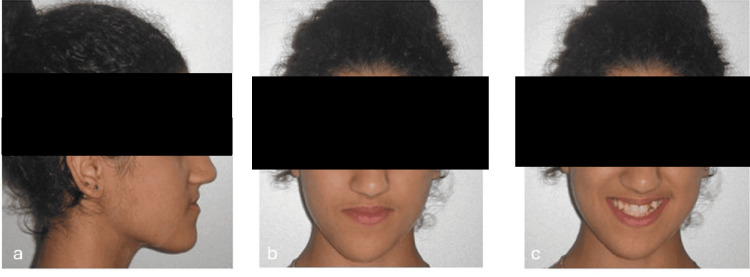
Extraoral photographs obtained before treatment. (a) Right profile view, (b) frontal view at rest, and (c) frontal smiling.

**Figure 2:**
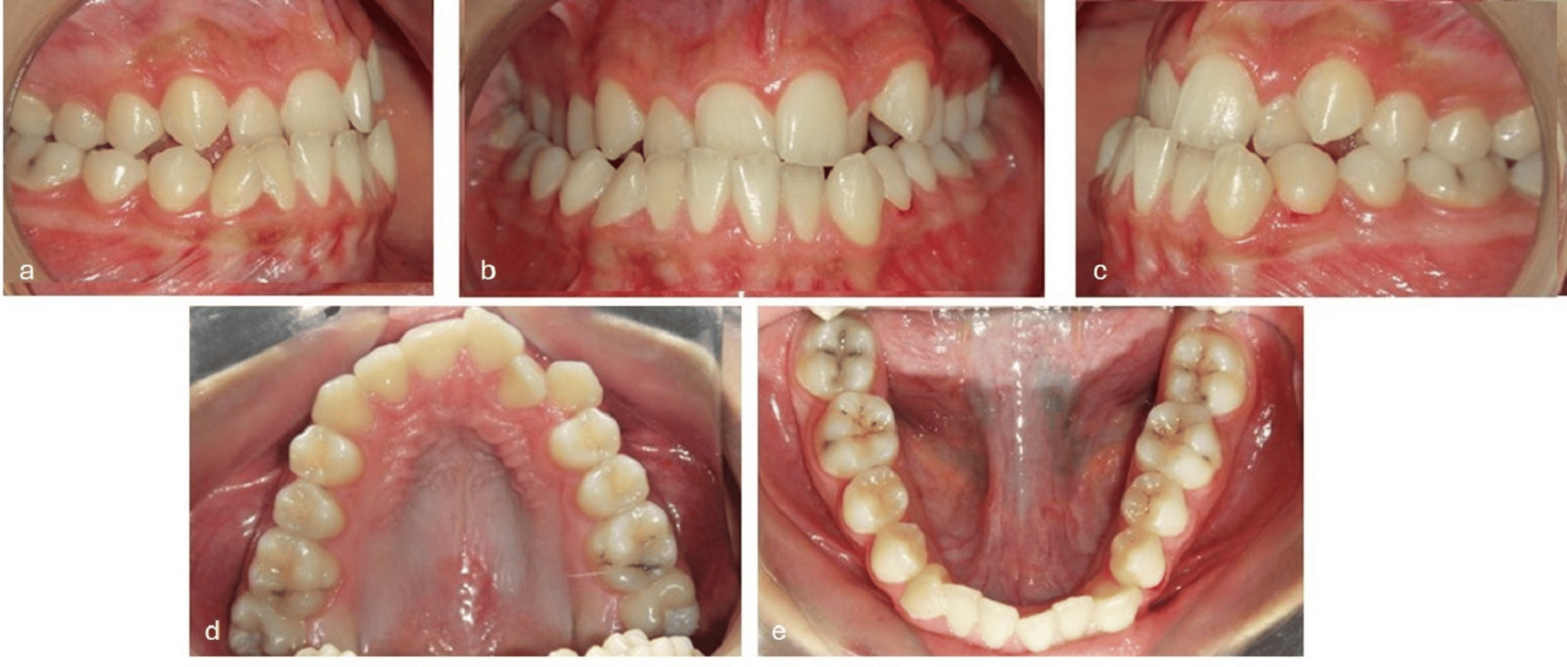
Intraoral photographs recorded at the pretreatment stage. (a) Right lateral view, (b) frontal view in maximum intercuspation, (c) left lateral view, (d) maxillary occlusal view, and (e) mandibular occlusal view.

**Figure 3:**
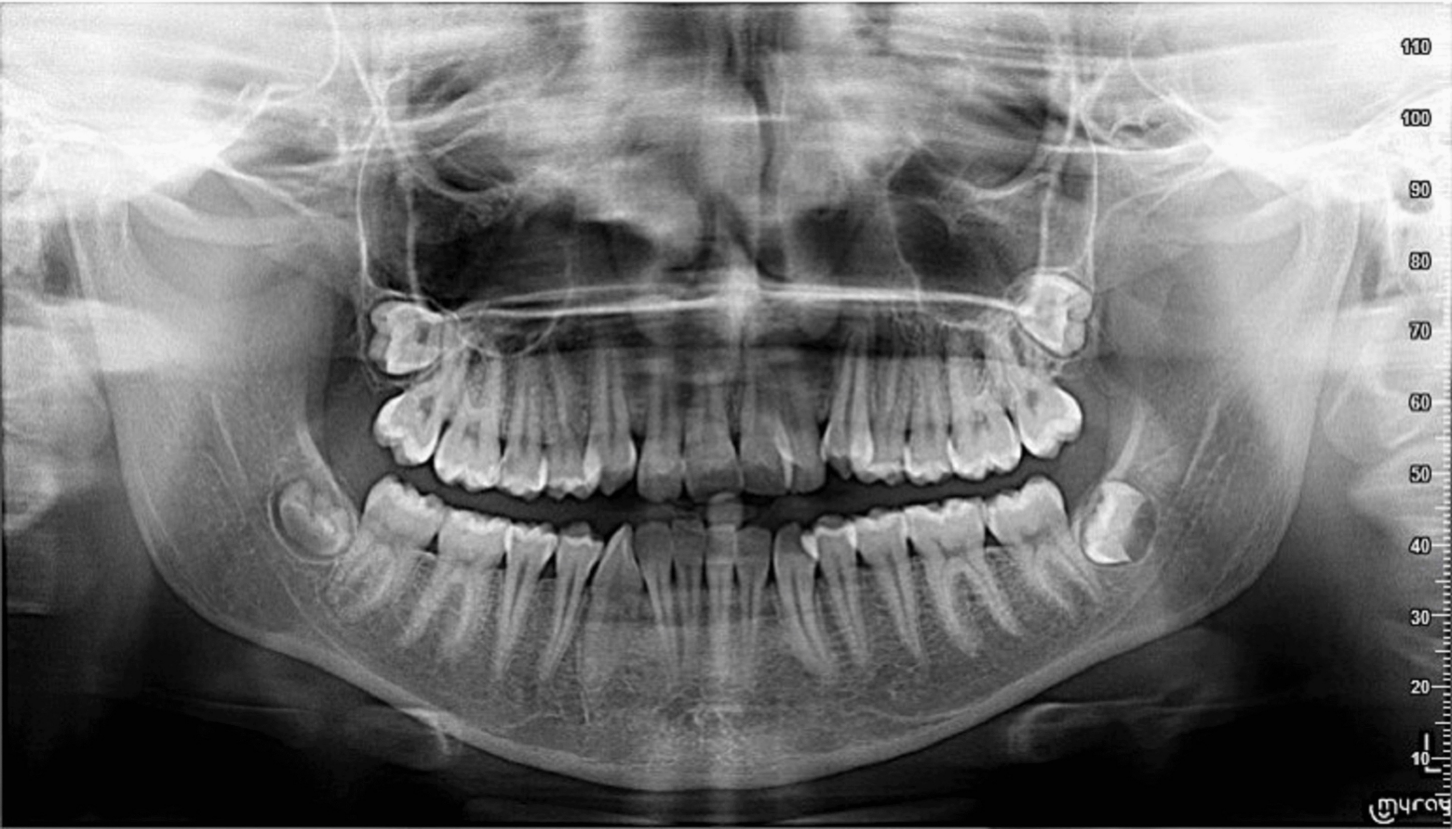
Panoramic radiograph taken before treatment. Panoramic radiograph showing the overall dentition and supporting structures.

**Figure 4:**
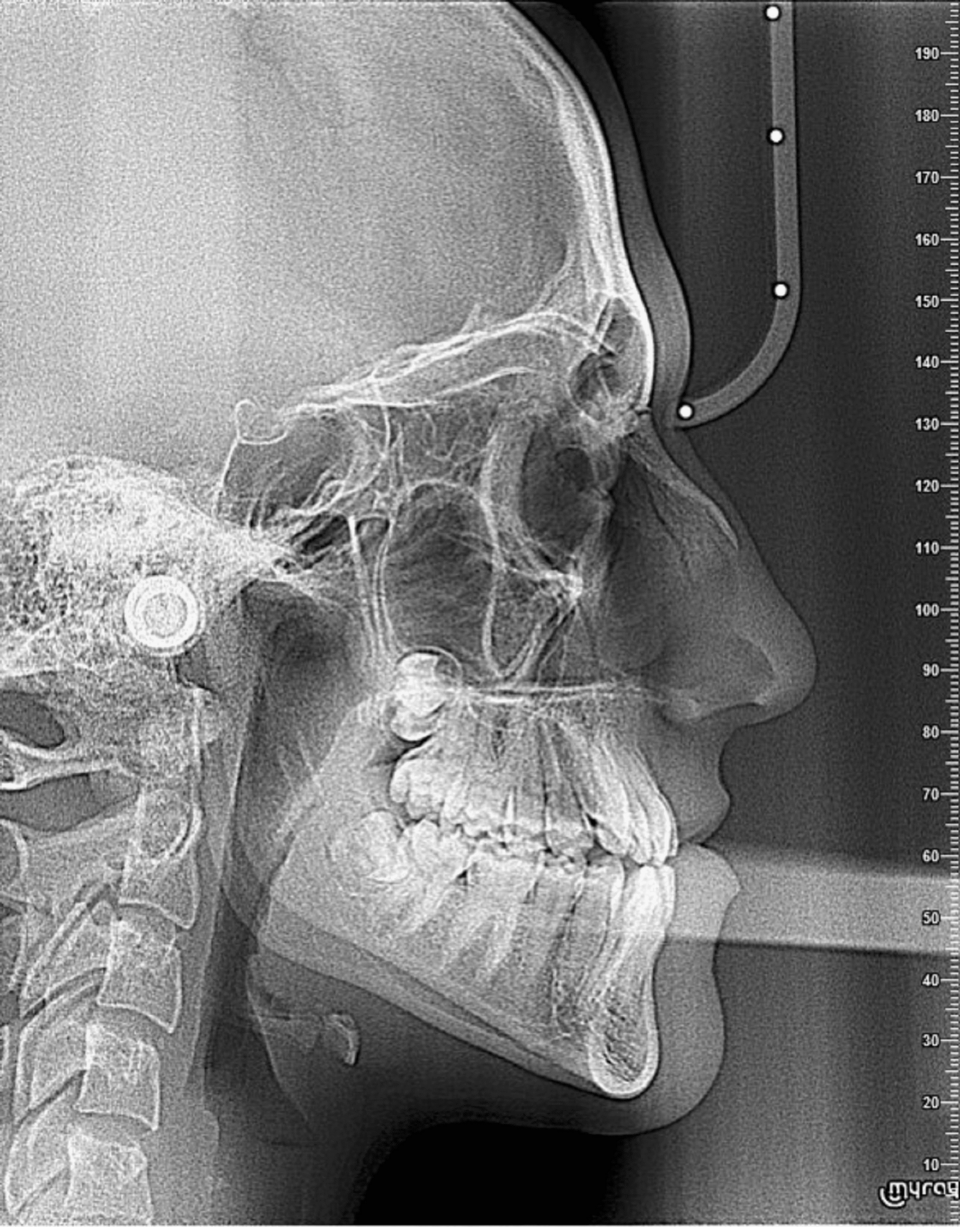
Lateral cephalometric radiograph taken before treatment. Lateral cephalometric radiograph illustrating the initial skeletal and dental relationships.

A 19-year-old female patient presented with the following primary complaint: “My lower teeth are in front of my upper teeth.” Clinical examination revealed a straight-to-concave facial profile with competent lips (Figure 1A), slight facial asymmetry, chin deviation to the right, and good vertical proportions (Figures 1B, 1C). A Class III occlusal relationship was noted on both sides (Figures 2A, 2C), accompanied by a 2 mm negative overjet and a 2 mm underbite (Figure 2B). Dental midlines were not coincident, having the mandibular midline displaced to the right, around 3 mm relative to the midline of the patient’s face. Occlusal analysis revealed approximately 6 mm of anterior maxillary crowding (Figure 2D) and 4 mm of anterior mandibular crowding (Figure 2E). The radiographic file comprised a panoramic radiograph and lateral cephalogram (Figures 3, 4). Cephalometric analysis (Table 1) showed a skeletal Class III malocclusion, a normodivergent vertical pattern with normal inclination of the lower incisors. All teeth were present.

**Table 1:** Cephalometric analysis. Pre- and post-treatment cephalometric values compared with established norms. SNA: Sella–Nasion–A Point Angle; SNB: Sella–Nasion–B Point Angle; ANB: A Point–Nasion–B Point Angle; FMA: Frankfort–Mandibular Plane Angle; IMPA: Incisor–Mandibular Plane Angle; FMIA: Frankfort–Mandibular Incisor Angle; U1-FH: Upper Incisor to Frankfort Horizontal Plane Angle; U1-L1: Upper Incisor to Lower Incisor Angle.

	Norm	Pre-treatment	Post-treatment
SNA	82°	89°	87°
SNB	80°	91°	88°
ANB	2°	-2°	-1°
Wits appraisal	0 mm	-12 mm	-7 mm
FMA	25°	23°	20°
IMPA	88°	87°	85°
FMIA	67°	75°	70°
U1-FH	107°	116°	117°
U1-L1	135°	133°	127°
Occlusal plane	10°	1°	7°
Z angle	75°	77°	76°
Upper lip	mm	10	9
Total chin	mm	10	8

Treatment goals

A combined surgical-orthodontic approach was initially proposed as the most appropriate treatment option, but was declined by the patient due to concerns regarding the cost and invasiveness of the surgery. Thus, the adopted treatment plan was to treat the Class III malocclusion by mandibular molar distalization using microimplant anchorage through a direct anchorage protocol, control the vertical dimension and lower incisors inclination, and establish a bilateral Class I occlusion.

Treatment procedure

**Figure 5:**
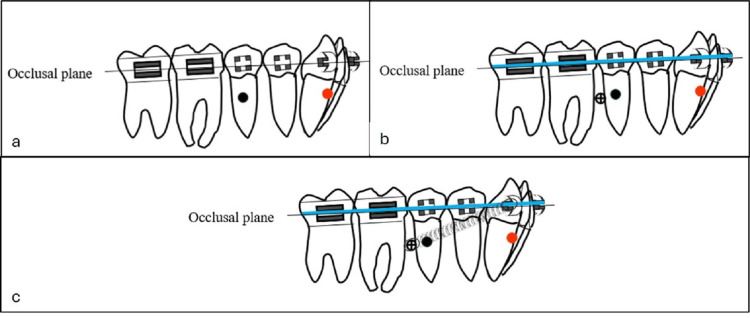
Schematic illustration showing the biomechanics used for lower molar distalization and occlusal plane control. (a) Initial appliance configuration with 0.022 × 0.028-in brackets. The mandibular arch was bonded from the right second molar to the left second molar. The black circle indicates the center of resistance of the entire mandibular arch, whereas the red circle represents the center of resistance of the anterior segment. (b) After the leveling and alignment stage, a 0.019 × 0.025-in stainless steel archwire was inserted. A microimplant (indicated by a crossed circle) was placed bilaterally between the second premolar and first molar. (c) Bilateral nickel–titanium (NiTi) coil springs were connected directly from the microimplant to the canine to deliver distalizing forces. Source: Figure created by the author using PowerPoint (Microsoft Corporation, Redmond, WA).

**Figure 6:**
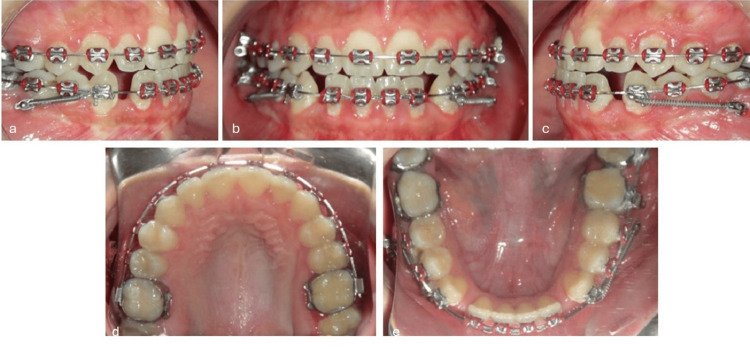
Intraoral photographs obtained during the course of treatment. (a) Right lateral view, (b) frontal view in maximum intercuspation, (c) left lateral view, (d) maxillary occlusal view, and (e) mandibular occlusal view.

MBT-prescription 0.022” brackets (Mini Uni-Twin, 3M, Monrovia, CA) were bonded on the lower arch, bypassing the incisors at the first stage to prevent their proclination (Figure 5A). At six months of treatment, after leveling and alignment of both arches using 0.019” x 0.025” stainless steel archwires, 1.4 mm x 8 mm microimplants (AbsoAnchor, Dentos, Inc., Daegu, Korea) were placed under local anesthesia bilaterally between the roots of the second premolar and the roots of the first molar at an angle of 30° to the teeth axis to maximize cortical bone engagement (Figure 5B). NiTi coil springs were activated from the microimplants to the lower canines on both sides (Figure 5C) to deliver approximately 180 g of force per side, gauged with a dynamometer. Subsequently, the mandibular incisors were bonded and thus retracted. During treatment, the maxillary arch was expanded and bonded (Figure 6D), while the mandibular occlusal view demonstrated progressive alignment and leveling of the lower arch with maintenance of anterior space through fixed appliance mechanics (Figure 6E). Twelve months later, the lower dentition was distalized by approximately 6 mm per side into a Class I canine and molar (Figures 6A-6C). Treatment was completed after 18 months, retainers were delivered, consisting of fixed and wraparound retainers on both arches, then the patient was sent for third molars removal.

Treatment outcomes and evaluation

**Figure 7:**
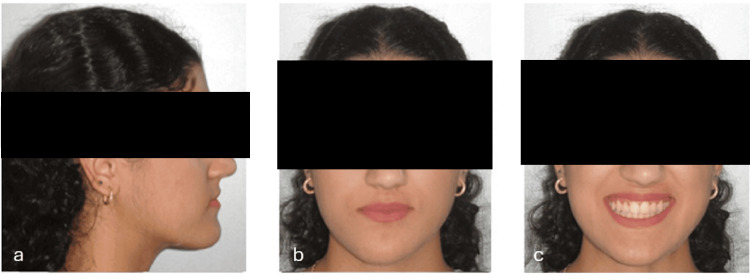
Extraoral photographs taken after completion of treatment. (a) Right profile view, (b) frontal view at rest, and (c) frontal smiling.

**Figure 8:**
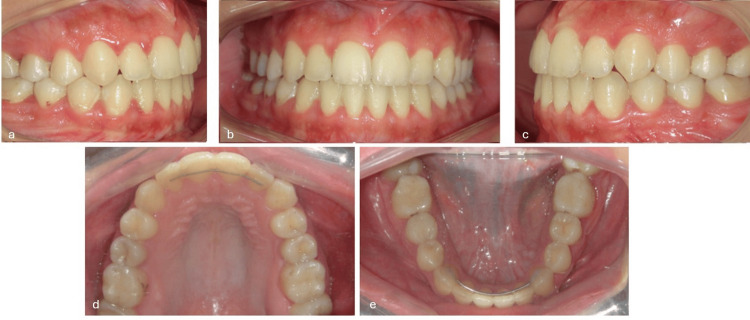
Post-treatment intraoral photographs. (a) Right lateral view, (b) frontal view in maximum intercuspation, (c) left lateral view, (d) maxillary occlusal view, and (e) mandibular occlusal view.

**Figure 9:**
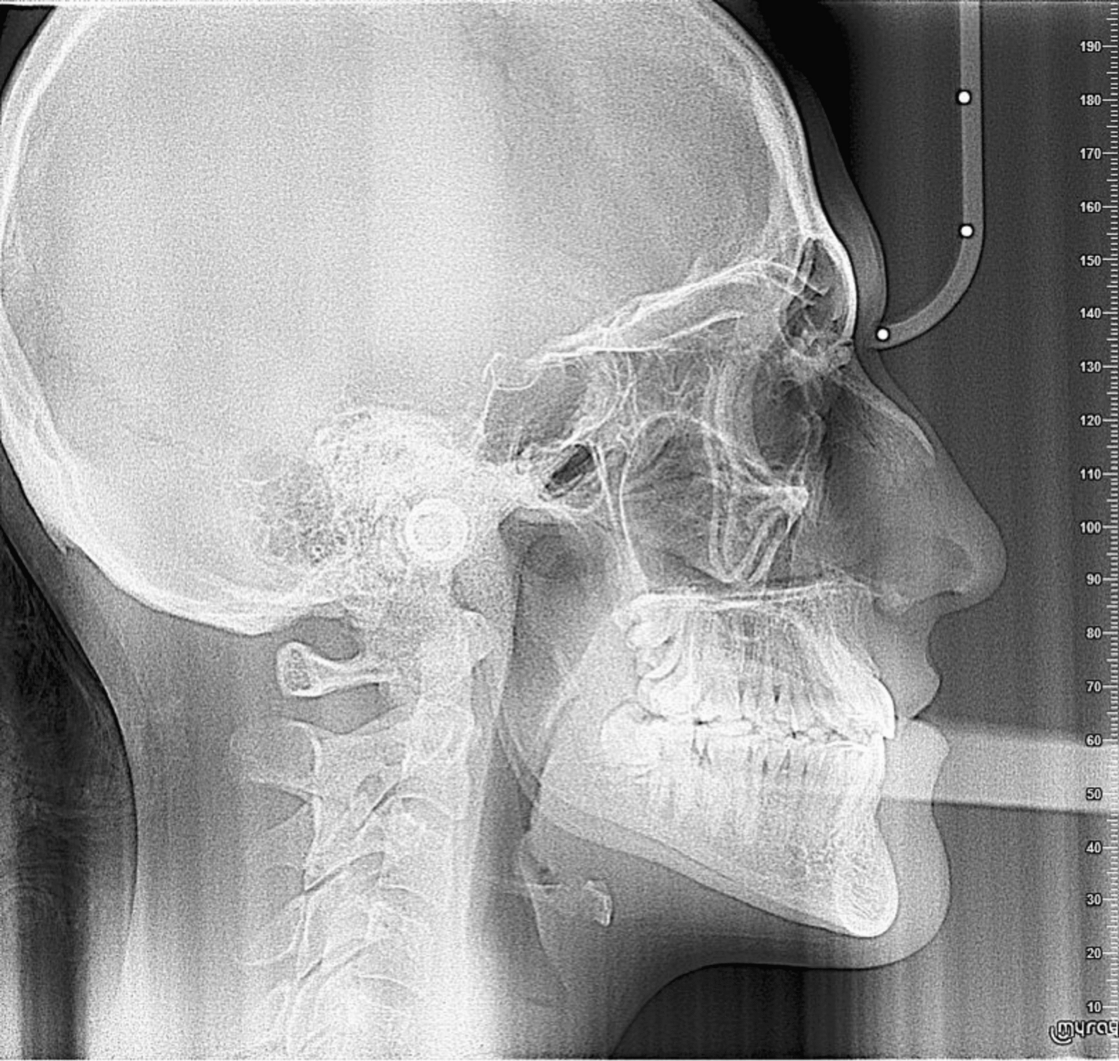
Lateral cephalometric radiograph taken after treatment. Lateral cephalometric radiograph demonstrating the final skeletal and dental relationships.

**Figure 10:**
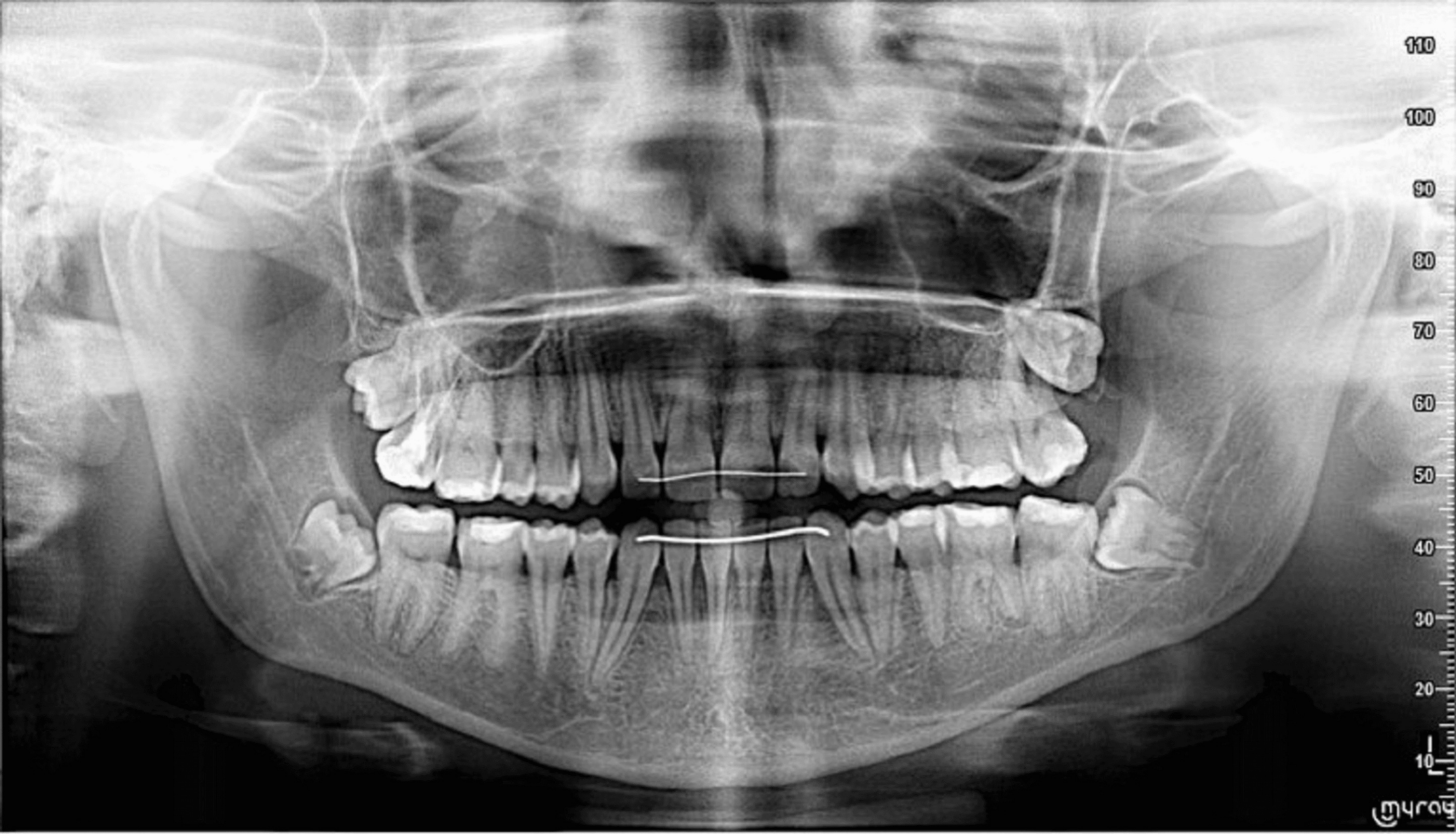
Panoramic radiograph obtained after treatment. Panoramic radiograph demonstrating overall alignment and root parallelism.

**Figure 11:**
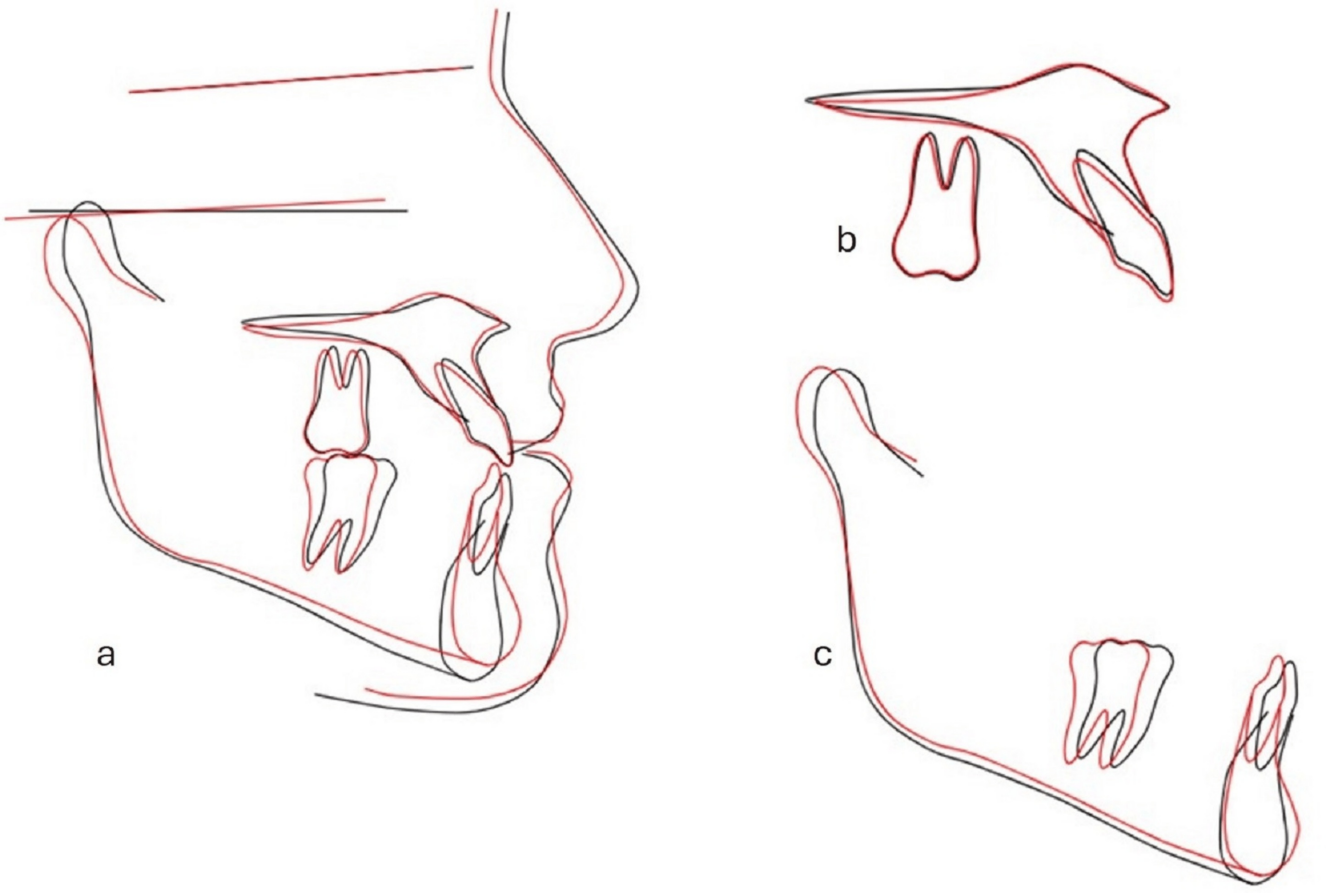
Superimposition of the pre-treatment and post-treatment lateral cephalometric tracings. (a) Overall superimposition on the anterior cranial base, (b) maxillary regional superimposition showing positional changes of the maxillary incisors and molars, and (c) mandibular regional superimposition showing positional changes of the mandibular incisors and molars.

Post-treatment extraoral pictures showed an enhanced profile (Figure 7A) with enhanced mandibular projection and a remaining slight chin divergence to the right, which had been explained at the outset as a possible outcome in the absence of surgical treatment (Figure 7B, 7C). An ideal occlusion was established, achieving a Class I canine and molar on both sides (Figures 8A, 8C) with corresponding midlines (Figure 8B). Overjet was corrected to +2 mm, and overbite was normalized to 2 mm (Figure 8B). Post-treatment maxillary and mandibular occlusal views showed well-aligned arches with resolution of the previous anterior crowding and harmonious arch forms (Figures 8D, 8E). Post-treatment panoramic and lateral cephalogram (Figures 9, 10) showed adequate root parallelism, no root resorption, and cephalometric values within the normal range (Table 1). Cephalometric superimposition demonstrated distalization of the lower dentition and minimal anchorage loss. A rotation of the mandibular plane in a counterclockwise direction was observed, which promoted anterior bite closure and, most importantly, the control of the vertical dimension with no extrusion of the posterior teeth (Figure 11). Lower incisors exhibited slight proclination while maintaining their position within the mandibular symphysis envelop.

**Figure 12:**
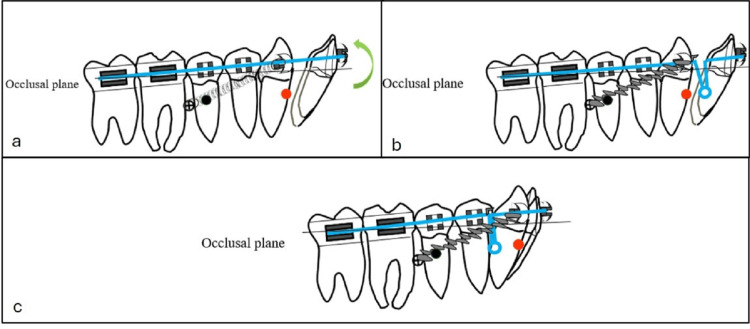
Schematic illustration showing the biomechanics used for lower molar distalization and occlusal plane control. (a) At the completion of mandibular arch distalization, a counterclockwise rotation of the mandibular arch (green arrow) was observed as a consequence of direct skeletal anchorage. This movement contributed to anterior bite closure and improved vertical control without extrusion of the posterior teeth. (b) Retraction of the incisors carried out using loop mechanics, with the posterior segment stabilized to the microimplant by a steel ligature tied to the canine. (c) Final stage following completion of incisor retraction. Source: Figure created by the author using PowerPoint (Microsoft Corporation, Redmond, WA).

Furthermore, closing loops were incorporated into the mandibular archwire to achieve controlled, frictionless retraction of the lower incisors while maintaining posterior anchorage (Figures 12B, 12C). Mandibular molar distalization, supported by micro-implant anchorage, relies on precise modulation of the force vector to control occlusal plane rotation and achieve bodily distal movement of the mandibular dentition (Figure 12).

## Discussion

Successful mandibular molar distalization requires precise biomechanical planning [7]. Effective tooth movement depends not only on the application of force but also on factors such as force magnitude, direction, anchorage design, and the relationship between the center of resistance of the dental arch and the applied force [8].

Traditional distalization mechanics often produce limited posterior movement and may cause an undesirable rotation of the mandibular plane in a clockwise manner. This vertical change can negatively influence occlusion and facial proportions, making the correction of Class III malocclusion more difficult [9].

The use of temporary anchorage devices has made controlled distalization more predictable by eliminating the need for dental anchorage [10]. Numerous studies have described different mechanical protocols involving variations in screw placement, force levels, and archwire configurations.

Although concerns have been raised regarding possible complications such as root proximity or soft-tissue irritation, interradicular placement remains one of the most commonly used sites for microimplant insertion [11,12]. Several investigations have suggested that this location can provide effective distalization with minimal tipping when appropriate force vectors are applied [1]. In fact, the force vector should ideally be oriented parallel to the occlusal plane and positioned close to the center of resistance of the posterior segment [7,13]. Auxiliary devices such as power arms may help achieve this configuration [2,4].

In the present case, distalization was achieved using direct anchorage mechanics, with NiTi coil springs connecting mandibular canines to microimplants. NiTi coil springs were selected because they deliver relatively constant and light forces compared with elastomeric chains, which are prone to rapid force decay.

Most authors recommend forces ranging between 150 and 200 grams per side for en-masse mandibular distalization. Excessive forces may jeopardize microimplant stability and lead to undesirable tooth movement or occlusal disturbances [14-16].

The applied mechanics produced a counterclockwise rotation of the mandibular plane, resulting in anterior bite closure and improved vertical control without posterior extrusion (Figure 12A). Such mechanics are particularly advantageous in patients with normodivergent or hyperdivergent facial patterns [17].

After achieving a Class I canine relationship, the mandibular incisors were retracted using loop mechanics while maintaining posterior anchorage with the microimplants (Figures 12B, 12C). Distalization was successfully achieved despite the presence of the mandibular third molars, as their buds were positioned low and did not interfere with the distalization process. Class III elastics were avoided to prevent extrusion and retroclination of the lower incisors.

Radiographic evaluation confirmed preservation of the alveolar bone surrounding the mandibular incisors [16]. While some studies have reported reductions in alveolar bone thickness following distalization, the findings in the present case were consistent with reports describing adaptive bone remodeling in the symphyseal region [18,19].

In our case report, the reliance on two-dimensional imaging constitutes a limitation. A three-dimensional evaluation would provide a more precise assessment of alveolar bone changes.

## Conclusions

This case report demonstrates that direct skeletal anchorage using interradicular microimplants can be an effective non-surgical option for the camouflage management of skeletal Class III malocclusion. When mandibular distalization as well as vertical control are required, this biomechanical approach can produce favorable occlusal and facial outcomes through controlled posterior tooth movement and counterclockwise rotation of the mandibular plane.

Careful case selection and accurate biomechanical planning remain essential for achieving predictable results. In our next case report, we will demonstrate how an indirect anchorage distalization protocol can achieve the desired outcomes and identify the types of patients in whom it may be indicated. Future studies incorporating three-dimensional imaging techniques such as cone-beam computed tomography may further clarify the extent of alveolar remodeling associated with this treatment modality.
